# Assessment of Muscular Strength and Functional Capacity in Smoker Population Without Any Diagnosed Respiratory Disease: A Cross-Sectional Study

**DOI:** 10.3390/healthcare13050493

**Published:** 2025-02-24

**Authors:** Christophe Thibon, Gilles Caty, Sophie Gohy, Frank Aboubakar Nana, Gregory Reychler

**Affiliations:** 1 Secteur de Kinésithérapie et Ergothérapie, Cliniques Universitaires Saint-Luc (UCL), Avenue Hippocrate 10, 1200 Brussels, Belgium; gregory.reychler@uclouvain.be; 2 Institut de Recherche Expérimentale et Clinique (IREC), Pôle de Pneumologie, ORL & Dermatologie, Université Catholique de Louvain, 1200 Brussels, Belgium; sophie.gohy@uclouvain.be (S.G.); frank.aboubakar@uclouvain.be (F.A.N.); 3 Service de Médecine Physique et Réadaptation, Centre Hospitalier Wallonie Picarde (CHWAPI), 7500 Tournai, Belgium; gilles.caty@chwapi.be; 4 Service de Pneumologie, Cliniques Universitaires Saint-Luc (UCL), 1200 Brussels, Belgium

**Keywords:** functional capacity, muscle, exercise, physical capacity, tobacco, sit to stand

## Abstract

**Introduction**: Smoking is a risk factor for chronic obstructive pulmonary disease (COPD) and lung cancer. In addition to pulmonary damages, peripheral muscle impairments are present in this population. Pulmonary limitation is observed in smokers before disease diagnosis, but functional capacity limitations are uncertain, contrary to patients who have already been diagnosed. The aim of this study was to compare muscular strength and endurance between non-smoker and smoker populations without any diagnosed respiratory disease. **Method**: This cross-sectional study assessed subjects without diagnosed respiratory disease in terms of physical capacity using two tests (one-minute sit-to-stand test (STST) and Jamar dynamometer test (JDT)). **Results**: The sample consisted of 147 subjects. The number of repetitions and the muscle strength were lower in the smoker than in the non-smoker population (28.5 ± 8.7 vs. 33.5 ± 8.2 (*p* < 0.001), and 38.2 ± 10.1 vs. 42.4 ± 10.5 (*p* = 0.04), respectively). The relative change in heart rate during STST was lower in the smokers compared to the non-smokers (*p* = 0.01). No significant differences were found based on gender. **Conclusions**: Smokers without alcohol or drug dependence and without diagnosed lung disease exhibit non-clinically but statistically reduced muscular strength and endurance. Their heart rate response to exercise is also reduced.

## 1. Introduction

Despite many prevention campaigns, 1.3 billions of people are still smoking tobacco (WHO). Smoking is an important concern due to its role in the appearance of diseases. Indeed, tobacco smoke is the first risk factor of chronic obstructive pulmonary disease (COPD), and its association with lung cancer has been well established for a long time [1]. In the world, 10–15% and 20–30% of smokers develop lung cancer or a COPD, respectively [2].

Recently, the focus has been on subjects without a diagnosed lung disease but at risk of developing one (pre-*COPD* [3] and *PRISm* (Preserved Ratio Impaired Spirometry) [4]). Many of these subjects are or were smokers and have already experienced typical symptoms of respiratory diseases. In a large population-based cohort study (n = 4572), 63 and 69% of the subjects who had normal spirometry and PRISm results, respectively, were smokers.

Patients with diseases related to tobacco frequently present muscle atrophy [5] and peripheral muscle impairment [6]. Impairment was shown to be lower in age-matched controls than in patients with a respiratory disease [5], but the tobacco, gender, and body mass index, which are well-known risk factors, were not considered in the matching. Indeed, free radicals or reactive oxygen species contained in the cigarettes were demonstrated to be detrimental to skeletal muscle [7], with resulting oxidative damages observed in the muscle proteins of smokers without lung disease [8]. Muscle dysfunction could result in a reduced functional capacity.

We hypothesized that smokers may experience an early limitation in muscular strength and functional exercise capacity and that this could be influenced by gender. Indeed, men and women are not similarly impacted by tobacco smoke. Smoking patterns and their effects on the neurological system vary by gender [9]. These differences may be attributed to genetic predisposition or hormonal variations between genders [10,11].

The aim of this study was to assess muscular strength and endurance among a smoker population without any diagnosed respiratory disease. There are limited studies in this area. If smokers were found to exhibit lower muscular strength and endurance, physical capacity assessments could be incorporated into smoking cessation services, and smokers could be encouraged to engage in regular physical activity through tailored programs.

## 2. Method

### 2.1. Study Design

This study was a cross-sectional study and followed the “STrengthening the Reporting of OBservational studies in Epidemiology” (STROBE) guidelines.

### 2.2. Subjects

Voluntary subjects were randomly recruited at a train station. They had to accept to participate after oral invitation. The inclusion criteria were an age between 40 and 70 years and being able to understand the explanation of the different tests. The exclusion criteria included suffering from a diagnosed respiratory disease, being under the influence (alcohol, psychotropic drugs), suffering from any disease or situation that could interfere with the results (orthopedic pathology, degenerative illness), and being a former smoker. After inclusion, the subjects were divided in two groups based on their smoking status: active smokers (defined as individuals who smoked at least one cigarette per day, every day) versus non-smokers.

Subjects who accepted to participate in this study completed a preclinic questionnaire to collect their demographic data, number of packs per year, and any eventual disease or treatment, alongside the CAGE questionnaire (Cut down drinking, Annoyed by criticism, Guilty feelings, and Eye-opener) to assess substance influence. The latter has been validated as a screening tool [12] and consists of four questions related to alcohol use. Item responses are scored (1 point for “yes” answers): the higher the score, the greater the indication of alcohol problems. The normal cutoff for CAGE scores is two positive answers [13].

Ethical approval for this study was obtained from the local ethics committee of the Cliniques universitaire Saint Luc and Université Catholique de Louvain in Brussels (B403201836437), prior to its start. All subjects included signed a written informed consent form. This study was conducted according to the declaration of Helsinki and respected Good Clinical Practice during all experimentations.

### 2.3. Settings

Experimentations were conducted from August 2018 to March 2019 by one qualified physiotherapist. The physical capacity assessment was based on two tests, carried out in a random order to avoid any influence. Before each test, explanations and a demonstration were given to the subject.

### 2.4. Tests and Outcomes

#### 2.4.1. The One Minute Sit-To-Stand Test (STST)

The STST has been previously validated for assessing functional exercise capacity and muscular strength [14] with a standardized chair (46 cm high, without arm rest) [15]. Subjects were instructed to stand up from the chair to a total-extension position and sit back as many times as possible within one minute. Arms had to remain crossed on the chest. Standardized instructions were provided, but no encouragement was given. Subjects performed two attempts with a 20 min rest period between each attempt [16]. The first attempt was considered as a training test. The number of completed repetitions during the STST was recorded and expressed in absolute and relative values. The relative values were calculated based on the equation proposed by Strassman et al. [17] (58.64 + 3.31 × gender (0 = female; 1 = male) − 1.84 × age − smoking status (0.96 = ex-smoker; 3.14 = smoker). Cardiorespiratory parameters such as the heart rate and pulsed oxygen saturation were recorded before and after the STST (NONIN, Onyx, Plymouth, Minnesota), and they were expressed in absolute values and change from baseline. A desaturation was considered when the pulsed oxygen saturation decreased by more than 4%. Initial heart rate and pulsed oxygen saturation were expected to be recovered before each new test.

#### 2.4.2. The Hand Grip Test (HGT)

The Jamar dynamometer was used to assess the isometric grip strength expressed in kilograms. Subjects were seated, their shoulders in a neutral position, elbow flexed at 90°, and wrist positioned with 0 to 30° of dorsiflexion. The arm was not supported [18]. Subjects performed three attempts with one minute of rest between each attempt. The best of the three results of strength measurement was selected for analysis, and the result was expressed in absolute and relative values based on the equation proposed by Wang et al. [19], as follows: for male subjects, −29.959 − 3.095 × 10^−5^ × (age^3^ (years) + 38.719 × (height (m)) + 0.113 × (weight (kg));

and for female subjects, −22.717 − 1.920 × 10^−5^ × (age^3^ (years)) + 30.360 × (height (m)) + 0.048 × (weight (kg)).

### 2.5. Statistical Analysis

Data were computed using SPSS 25.0 (SPSS Inc., Chicago, IL, USA) and EasyMedStat 3.37.1 for Windows. To address potential biases in the baseline characteristics between groups, propensity score-matching with replacement was performed, allowing us to match the same control patient to several patients in the treatment group. Propensity scores were calculated using a logistic regression model [20], including the following normalized covariates: age, height, weight, and gender. They were selected because they are influencing factors on muscles or the sit-to-stand test. The covariates had no missing data. Non-smokers were matched to smokers by the nearest neighbor 1:1 matching algorithm, with a caliper value of 0.1 of the pooled standard deviation of the logs of the propensity score. It paired each patient in the treatment group with patients from the control group who shared the closest propensity score value [21]. Standardized mean differences (SMD) before and after matching were calculated to compare the baseline characteristics after matching. A post-matching SMD below 0.1 was considered an acceptable difference. The common support assumption was assessed using the Kolmogorov–Smirnov nonparametric test. Common support intervals were determined using the trimming method and kernel density estimators to maximize precision without worsening bias. The threshold was set at 0.001.

Tests results were compared between moderate and heavy smokers, according to the number of smoked packs per years (arbitrary cutoff > 10 packs per years (20 cigarettes by pack)) and between men and women, using Student’s *t*-test because of the central limit theorem. Correlation between data was analyzed using Pearson (r) correlation coefficients because of the central limit theorem. The strength of the correlation was interpreted as follows: 0 to 0.19 = very weak correlation, 0.20 to 0.39 = weak correlation, 0.40 to 0.59 = moderate correlation, 0.60 to 0.79 = strong correlation, and 0.80 to 1.0 = very strong correlation [22]. We used the Chi-Square test to compare the proportions. A *p*-value lower than 0.05 was considered statistically significant.

## 3. Results

### 3.1. Participants

Out of the 758 individuals invited to participate in this study, a total of 553 individuals declined participation, 56 did not meet the inclusion criteria, and 2 were unable to perform the tests. The final sample consisted of 147 subjects (101 smokers vs. 46 non-smokers), representing 19.4% of those initially approached.

The gender distribution was similar between the two recruited groups (*p* = 0.60). The mean number of packs per years was 17.8 ± 8.9. Non-smokers tended to be more overweight compared to smokers (67.3% vs. 51.5% had a BMI > 25, *p* = 0.07). The smokers were younger (mean age = 51 ± 8.5 y.o. vs. 54 ± 7.6 y.o., *p* = 0.04) and lighter (mean weight (kg) = 75.3 ± 14.9 vs. 81.8 ± 16.2, *p* = 0.02) than the non-smokers. The median CAGE score was below 2 and similar in both groups (1.12 ± 1.3 vs. 0.85 ± 1.0, *p* = 0.16). The participants reported chronic conditions such as hypertension (14 vs. 15%, *p* = 0.82), type 2 diabetes mellitus (2 vs. 2%, *p* = 0.86), and depression (5 vs. 5%, *p* = 0.98), with comparable prevalence and treatment rates between smokers and non-smokers.

After propensity score-matching, 61 subjects were included in the two groups (60.4% of the subjects from the initial group of smokers), with no differences between the four included covariates (age, height, weight, and gender). The demographic parameters of the subjects after propensity score-matching are displayed in Table 1.

### 3.2. Tests

The tests results are summarized in Table 1. The number of repetitions during the STST was lower in smokers (absolute values: change = 3.5, *p* < 0.001; relative values: change = 10.8%, *p* < 0.001). At the baseline, smokers had a higher heart rate compared to non-smokers (86 ± 12 bpm vs. 77 ± 11 bpm, *p* = 0.004). The relative change in heart rate during STST was lower in the smoker group compared to the non-smoker group (*p* = 0.01). At the baseline, both groups showed similar and normal pulsed oxygen saturation (mean = 97%, *p* = 0.13). Only one subject had a pulsed oxygen saturation inferior to 94% at the baseline. The mean change in pulsed oxygen saturation during the STST was lower than 4% and similar in both groups (*p* = 0.13). Four subjects in the smoker group and one in the non-smokers presented oxygen desaturation during STST.

When the STST results were compared between smokers and non-smokers, separately for men (n = 35 vs. 43) and women (n = 26 vs. 18), no significant differences were found for the absolute (change men = 2 (*p* = 0.35) and change women = 2.7 (*p* = 0.11) for smokers vs. non-smokers, respectively) and relative values (change men = 6.2% (*p* = 0.15) and change women = 6.9% (*p* = 0.22) for smokers vs. non-smokers, respectively). The difference in HR change observed between smokers and non-smokers in the overall group was only related to the male subgroups (45% vs. 34%, *p* = 0.02). The only significant correlation identified was a moderately negative relationship between STST performance and age (r = −0.43, *p* < 0.001). There were no significant correlations between the STST and other anthropometric parameters. Isometric grip strength was lower in the smoker group compared to the non-smoker group when expressed in absolute (change = 4 kg, *p* = 0.04) and relative values (change = 7.1%, *p* = 0.05). When comparing the grip strength between the smokers and non-smokers for men and women separately, a significant difference was observed for the absolute (change men = 4.6 kg, *p* = 0.02; change women = 1.6 kg, *p* = 0.32) and relative values (change men = 10.4%, *p* = 0.01; change women = 3.7%, *p* = 0.47) among men but not among women. The only significant correlation found between the HGT and the anthropometric parameters was a strong positive correlation with the weight (r = 0.64, *p* < 0.001).

**Figure 1:**
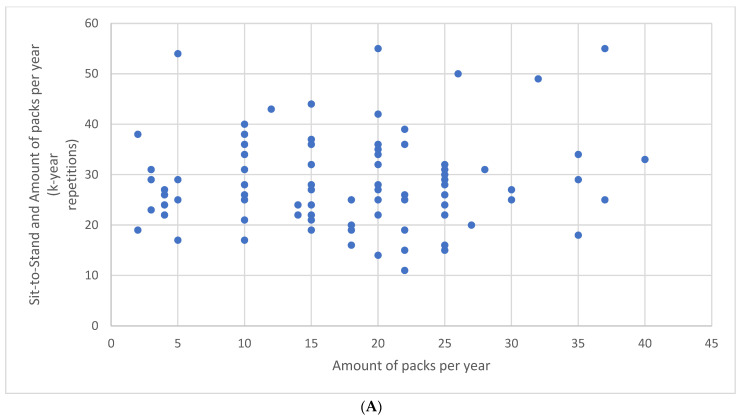

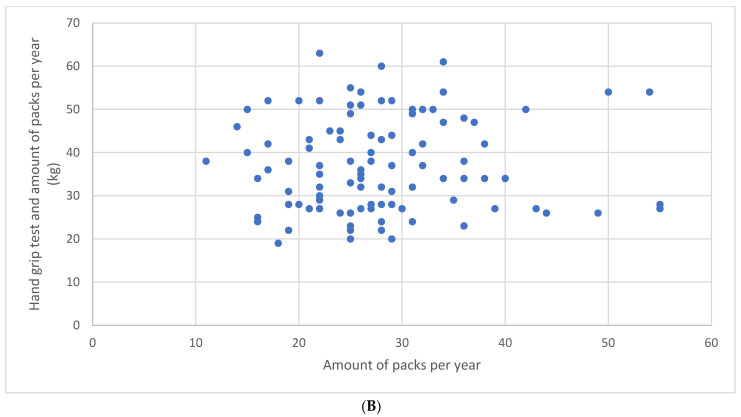
Relationship between the number of repetitions during the sit-to-stand test and the amount of packs per year (**A**) and between the hand grip test results and the amount of packs per year (**B**).

There was neither a correlation between the number of repetitions during the STST and the amount of packs per year (r = 0.06, *p* = 0.50) (Figure 1A) nor between the HGT results and the amount of packs per year (r = 0.04, *p* = 0.66) (Figure 1B).

We found no differences in STST performance (change = 2.5%, *p* = 0.50) or strength (change = 0.2 kg, *p* = 0.23) between moderate (n = 29) and heavy smokers (n = 72).

## 4. Discussion

This study investigated the muscular strength and functional exercise capacity among a smoker population without diagnosed lung disease. The results indicate that smokers exhibit reduced muscular strength and functional exercise capacity compared to non-smokers. In addition, the heart rate response to exercise was lower in the smoker group than in the non-smoker group, without differences in pulsed oxygen saturation.

The STST was validated (valid and reliable) to evaluate certain components of physical capacity in both the healthy and COPD population by measuring lower-limb muscular strength and functional exercise capacity [23]. The reduced number of repetitions observed during the STST indicated a lower physical capacity among the smoker population even though this decrease was not correlated with the number of packs per year. The STST results in the non-smoker group were better than in previous studies involving patients with COPD [15,24,25] but lower than in studies involving healthy subjects [17,26]. This difference could be attributed to an older age or variations in test application between studies. For instance, a “fully seated position” was required in our study, contrary to the only other study including smokers in which a touch-and-go method was accepted [17]. As expected, STST performance decreased with age [17]. Similarly to Strassman et al., who included smokers in their study where they determined the normal values for this test [17], our results confirmed that tobacco smoke had already had an effect on the subjects’ functional exercise capacity.

At the baseline, smokers had a greater heart rate than non-smokers, but this difference was expected, as it is a common effect of nicotine during the stimulation of sympathetic neurotransmission and then the heart [27,28]. The mean pulsed oxygen saturation was normal and similar in both groups. Even if cigarette smoke tends to be associated with reduced arterial oxygen saturation [29], a similar pulsed oxygen saturation is not surprising because pulse oximetry does not differentiate oxyhemoglobin and carboxyhemoglobin [30], while cigarette smoke is associated with an elevated rate of carboxyhemoglobin [31]. BMI and weight were lower among smokers, which is consistent with the literature [32]. No significant difference in pulsed oxygen saturation changes during the STST between the two groups was found. This finding suggests that the smoking status had no impact on the change in pulsed oxygen saturation during effort in smokers without disease. As expected, a lower heart rate response to exercise was observed in the smoker group. Tobacco smoke is known to impair the heart’s ability to increase its rate in response to exercise demands [27,28]. Indeed, a faster heart rate at rest, vasoconstriction [33], and inflammation-induced cardiovascular damages [34] impair the ability of the heart to respond to increased demands during exercise.

The HGT has been previously validated to assess upper-limb muscle strength in healthy subjects [35]. The values of the HGT in our non-smokers were similar to those previously obtained in other studies with non-smokers [19,36,37]. We found a significant difference in the isometric hand grip strength between smokers and non-smokers, and this difference was mainly attributed to the men in these groups. The detrimental effect of tobacco on skeletal muscle weakness remains mainly unexplained [38], but different hypotheses have been proposed regarding muscle wasting [39] resulting from systemic inflammation and reduced oxygenation due to tobacco, as well as the lower level of physical activity [40] and lean body mass [41] observed in smokers. Indeed, the women in our study did not show differences related to their smoking status. Two other studies assessed hand grip strength among smokers. One observed no difference in strength between smokers and non-smokers aged 40 to 70 years [42]. The other one demonstrated a lower grip strength among smokers, but the subjects were younger [43]. As expected, strength performance increased with weight [44].

We observed discrepancies when comparing smokers and non-smokers within the overall population and the separate groups of men and women. Both groups exhibited a similar trend, with smokers showing lower STST and HGT results, even though the comparison of the absolute and relative values was not statistically significant for most of them. This difference between gender subgroups and the overall population could be attributed to the small sample size when data for men and women were analyzed separately.

Interestingly, some outcomes differed between men and women. Heart rate response to exercise was only affected by tobacco among women, and hand grip strength was only impacted in men. These findings are in line with the literature, highlighting the different impact of tobacco on men and women [10].

The observed differences in hand grip strength and STST suggest that smokers may already exhibit reduced muscular strength and functional exercise capacity, regardless of the presence of disease. The lower hand grip strength recorded among our smoker population suggests that amyotrophy and structural muscular changes could begin before the appearance of a respiratory disease associated with tobacco [45]. However, the differences between smokers and non-smokers were not clinically relevant, as the MCID for the STST was three repetitions, and, for the HGT, it was from 5 to 6.5 kg [46,47]. This means that, even if we had observed a difference, the clinical impact would not have been relevant yet.

Several limitations need to be addressed. Although our results indicate diminished muscular strength and functional exercise capacity among smokers compared to non-smokers, a longitudinal study would be necessary to confirm these results by including confounding factors in the recruitment process. We did not assess the participants’ physical activity or sports habits, which could have influenced the results. It is possible that regular physical activity might have affected our findings, although the random recruitment method should have mitigated this influence. Moreover, some behavioral differences between smokers and non-smokers related to aerobic activities could have influenced the results by modifying some of the outcomes at the baseline. The number of subjects declining to participate was also a limiting factor. Subjects more educated or concerned with physical activity could have been more inclined to participate in this study. Thus, the proportion of subjects with limited functional capacity in our sample could be different compared to the general smoker population. While we focused on muscular strength and functional exercise capacity, which are critical components of physical capacity, it is important to note that physical capacity also encompasses cardiorespiratory fitness, flexibility, coordination, and balance [48]. Since we included only subjects older than 40 years old without alcohol or drug dependence and probably active in their daily life because of recruitment at a train station, the external validity of our study is limited to this demographic. Moreover, propensity score-matching included 60% of the initial data, which could have led to selection bias. Consequently, we cannot generalize that all smokers present a reduced physical capacity. It is also likely that younger smokers may perform more regular physical activity and have a better functional exercise capacity, despite no difference being observed in this area between the subcategories of smokers in our study. Some subjects potentially had non-diagnosed COPD, but these data could not be retrieved without a lung function test.

Smokers, even in the absence of respiratory or physical complaints, should undergo physical capacity assessments. Patients with subnormal physical capacity should be educated about the benefits of physical activity, similar to the approach used for oncologic patients [49]. In our study, a few smokers demonstrated severely limited physical capacity, with STST scores lower than those of patients with COPD undergoing pulmonary rehabilitation [25]. These individuals should be considered for referral to pulmonary rehabilitation programs.

## 5. Conclusions

Our results demonstrated that smokers without alcohol or drug dependence and without diagnosed lung disease exhibit non-clinically but statistically significant reductions in muscular strength and functional exercise capacity compared to a non-smoker population. A physical capacity test should be included in services to help people stop smoking. Furthermore, smokers should be systematically encouraged to practice regular physical activity, and assessment of a diminished physical capacity could lead to referral to specific exercise programs.

## Figures and Tables

**Table 1:** Subject characteristics and exercise capacity test results after propensity score-matching.

	Smokers	Non-Smokers	SMD	*p*
Male/Female	61 (35/26)	61 (43/18)	−0.28	0.19
Age (years)	52.2 ± 8.0)	53.4 ± 7.8	−0.16	0.503
Weight (kg)	77.2 ± 11.7	78.8 ± 12.8	0.13	0.348
Height (m)	1.73 ± 0.1	1.76 ± 0.1	−0.35	0.06
Sit-to-Stand Test				
Repetitions (number)	28.5 ± 8.7	33.5 ± 8.2		<0.001
Repetitions (% predicted value)	59.9 ± 16.9	71.4 ± 18.5		<0.001
HR change (%)	39 ± 1	47 ± 2		0.01
SpO2 change (%)	−0.7 ± 1.5	−0.3 ± 1.3		0.13
Hand Grip Test				
Strength (kg)	38.2 ± 10.1	42.4 ± 10.5		0.04
Strength (% predicted value)	103 ± 16	109 ± 16		0.05

(HR = heart rate; SpO2 = pulsed oxygen saturation).

## Data Availability

The data are available on demand to the corresponding author.

## Abbreviations

BMI	Body mass index
COPD	Chronic obstructive pulmonary disease
FEV1	Forced expiratory volume in 1 s
JDT	Hand grip test
STST	Sit-to-stand test
